# Arecoline Is Associated With Inhibition of Cuproptosis and Proliferation of Cancer-Associated Fibroblasts in Oral Squamous Cell Carcinoma: A Potential Mechanism for Tumor Metastasis

**DOI:** 10.3389/fonc.2022.925743

**Published:** 2022-07-07

**Authors:** Jinfei Li, Shuangyi Chen, Yuxuan Liao, Hongyi Wang, Dawei Zhou, Bo Zhang

**Affiliations:** ^1^ Department of Stomatology, Third Xiangya Hospital of Central South University, Changsha, China; ^2^ Xiangya School of Medicine, Central South University, Changsha, China

**Keywords:** cancer-associated fibroblasts, arecoline, cuproptosis, metastasis, oral squamous cell carcinoma

## Abstract

**Background:**

Metastatic disease remains the primary cause of death in patients with oral squamous cell carcinoma (OSCC), especially those who use betel nut. The different steps of the metastatic cascade rely on reciprocal interactions between cancer cells and the tumor microenvironment (TME). Cancer-associated fibroblasts (CAFs) are regarded as a significant component in the TME of OSCC. However, the precise mechanisms regulating CAFs in OSCC are poorly understood.

**Methods:**

Thirteen genes related to the arecoline were analyzed to explore the significant ones involved in arecoline-related OSCC metastasis. The GSE139869 (n = 10) and The Cancer Genome Atlas (TCGA)-OSCC data (n = 361) were mined for the identification of the differentially expressed genes. The least absolute shrinkage and selection operator (LASSO) regression was performed to identify the independent prognostic signatures. Gene Ontology (GO) and Kyoto Encyclopedia of Genes and Genomes (KEGG) analyses were conducted to explore the functional enrichment of selected genes, and gene set enrichment analysis of cuproptosis-related genes was completed. Spearman’s analysis and Tumor Immune Estimation Resource (TIMER) were used to visualize the correlation between the infiltration of CAFs and the gene expression. The correlation analysis of the cells and different genes, including CAF infiltration and transcripts per million expression, was assessed. The relationship between arecoline and CAFs was confirmed by cell counting kit-8 assay (CCK-8). CancerSEA was searched to identify the single-cell phenotype.

**Result:**

Arecoline-associated fibrosis-related OSCC differentially expressed genes (AFOC-DEGs), namely, PLAU, IL1A, SPP1, CCL11, TERT, and COL1A2, were screened out and selected from the Gene Expression Omnibus (GEO) database and TCGA database. AFOC-DEGs were highly expressed in OSCC, which led to poor survival of patients. Functional enrichment analysis, protein–protein interaction network construction, and Spearman’s correlation analysis all suggested that AFOC-DEGs were closely associated with cuproptosis. Cellular experiments demonstrated that arecoline stimulation could significantly increase the cell viability of CAFs. Single-sample Gene Set Enrichment Analysis (ssGSEA) results showed that GLS and MTF1 were highly expressed when fibroblasts proliferated at high enrichment levels. In addition, analysis of single-cell sequencing results suggested that OSCC cells with high expression of AFOC-DEGs were associated with OSCC metastasis.

**Conclusion:**

We found a close association between arecoline, cuproptosis, and CAFs, which might play an important role in the metastasis of OSCC.

## Introduction

Oral squamous cell carcinoma (OSCC) is the most common oral malignancy, and its increased prevalence in recent years has created a heavy burden on global health (1). According to the GLOBOCAN 2020, the number of cases of oral cancer in the world was estimated at 377,713, with an age-standardized incidence rate of 6.0 for men and 2.3 for women per 100,000 individuals. More importantly, oral cancer ranks the third most common malignancy in men [10.2 per 100,000) in low/medium-income countries (by Human Development Index (HDI)], which is highly related to betel chewing (2). As an aggressive oral cancer, OSCC possesses an important biological feature of early metastasis leading to a poor prognosis (3). Despite advances in early diagnosis and treatment, the 5-year survival rate for patients with OSCC is still 50.4%, and metastasis is the leading cause of death (4). As a unique part of the dietary culture in Asia, betel nut chewing is a causal factor of OSCC, and arecoline is considered to be the most important active constituent in betel nut (5, 6). Based on strong mechanistic evidence, arecoline is classified as a Group 1 carcinogen by the International Agency for Research on Cancer (7). Arecoline can induce squamous cell carcinoma in the mouse esophagus and tongue (8). Studies in human-related cell experiments also strongly demonstrate that arecoline possesses key carcinogen characteristics (9). Our previous research has shown that arecoline can promote the proliferation of fibroblasts in the oral mucosa and mediate oral mucosal fibrosis, a kind of precancerous lesion (10). However, the underlying biological effects of arecoline on cancer-associated fibroblasts (CAFs) in OSCC are not thoroughly illustrated.

Cell interactions within the tumor microenvironment (TME) are becoming increasingly recognized as having an essential role in tumor metastasis (11). Studies have proved that CAFs, the major component of the TME, are highly involved in the malignant progression of cancer (12–14). Utilizing biopsy materials from patients with OSCC, researchers have offered clinical clues that CAF appearance increases with enhanced tumor metastasis (15). In detail, CAFs can communicate with cancer cells and secrete cytokine-like molecules, including exosomes, contributing to tumor development, invasion, and metastasis (16). Similar to cancer cells, CAFs also undergo metabolism reprogramming induced by hypoxia and adjacent cancer cells (17). Enhanced glycolysis and weakened tricarboxylic acid (TCA) cycle are the key factors in regulating CAF proliferation (18, 19). Researchers found that arecoline could promote glycolysis leading to the increase of reactive oxygen species, which could inhibit the TCA cycle and mediate tumor metastasis (20, 21). Arecoline also led to a disordered progression of cellular aerobic metabolism by destroying cytoskeletal integrity and mitochondrial function (22). Interestingly, mitochondrial respiration components are also affected by copper such as the respiratory complex IV, cytochrome *c* oxidase, and the antioxidant enzyme superoxide dismutase 1 (23, 24). A recent study has also implicated the close relationship between copper and apoptosis (24). Given that there are no studies related to the cell death modes affected by arecoline in CAFs, it will be an interesting topic to explore the relationship between copper ionophore-mediated death (cuproptosis) in arecoline and CAFs.

In this study, we comprehensively analyzed the expression of arecoline-associated fibrosis-related OSCC differentially expressed genes (AFOC-DEGs) and their correlation with cuproptosis and CAFs of OSCC patients in the Gene Expression Omnibus (GEO) and The Cancer Genome Atlas (TCGA) databases. We also experimentally verified the relationship between arecoline and CAFs. Moreover, *via* CancerSEA, the correlation between AFOC-DEGs and single-cell cancer hallmarks was investigated. These findings shed light on the essential role of arecoline in OSCC metastasis and provide a potential relationship between arecoline, cuproptosis, and CAFs.

## Materials and Methods

### Data Collection and Processing

**Figure 1:**
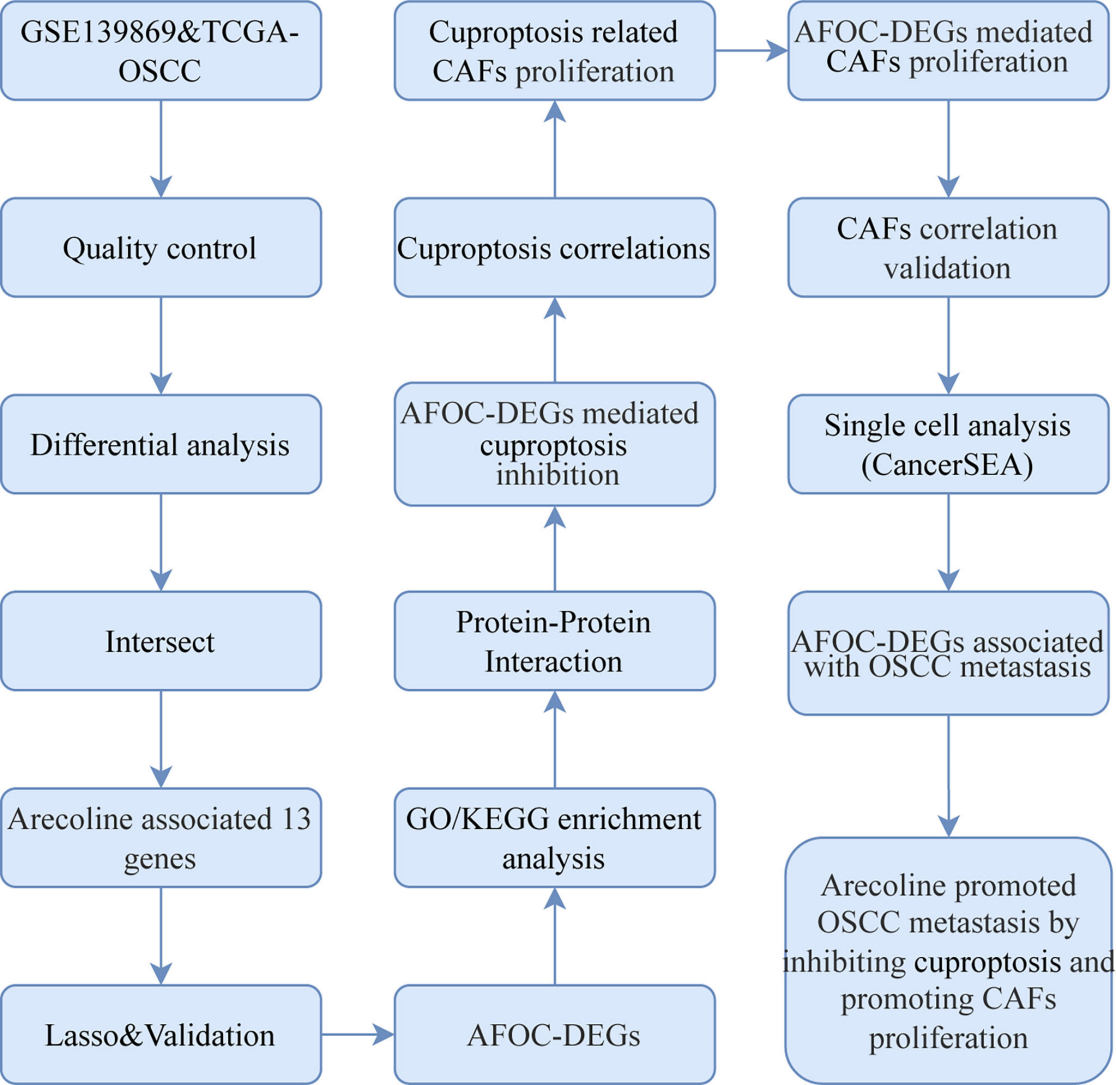
Flowchart for bioinformatics analysis of publicly available data from GEO, TCGA, and FibROAD databases. AFOC-DEGs, arecoline-associated fibrosis-related oral cancer differentially expressed gene; LASSO, least absolute shrinkage and selection operator; GO, Gene Ontology; KEGG, Kyoto Encyclopedia of Genes and Genomes; CAFs, cancer-associated fibroblasts; OSCC, oral squamous cell carcinoma; TCGA, The Cancer Genome Atlas.

High-throughput gene expression data of OSCC tissues and normal tissues were extracted from TCGA database (https://tcga-data.nci.nih.gov/tcga, TCGA-OSCC, n = 361, tumors = 328, normal = 3) (25), FibROAD (https://www.fibroad.org/download.php), and GEO Database (www.ncbi.nlm.nih.gov/geo; GSE139869, n = 10, tumors = 5, para-cancer = 5; GSE160395, n = 8, HSC-3 = 4, HSC-3-M3 = 4; GSE59414 and GSE38227, n = 7, arecoline = 4, control = 3) (26). the phenotypic correlation coefficient of the AFOC-DEGs was also analyzed from CancerSEA (http://biocc.hrbmu.edu.cn/CancerSEA/, Puram SV. *Cell*. 2017 (Oral cavity)). Our workflow for bioinformatics analysis of publicly available datasets from both the GEO and TCGA databases is illustrated in **Figure 1**.

### Differential Gene Analysis and Construction

In order to identify the DEGs, the limma R package was applied to compare the microarray expression profiles of OSCC tissues from GEO. Meanwhile, the batch effects caused by data merging were removed with the limma R package while analyzing expressions of cuproptosis-related genes after arecoline treatment. DESeq2 package was applied to compare the RNAseq expression profiles of OSCC tissues from TCGA after normalization. To normalize the gene expression from TCGA, the fragments per kilobase per million (FPKM) was converted to transcripts per million (TPM) using the following formula:


$$TPM_{i}=\frac{FPKM_{i}}{Total\;library\;FPKM}\times 10^{6}$$

The |Log_2_Fold Change| > 1 and adjusted p-value (false discovery rate (FDR)) < 0.5 were considered as the threshold values for DEG identification. The volcano plot and heatmap were performed with the “ggplot2” package of R software. The Venn diagram was then plotted to illustrate the union target genes of three gene sets.

### Evaluation of the Prognostic Value of Differentially Expressed Genes

To assess the prognostic value of the DEGs from TCGA, the least absolute shrinkage and selection operator (LASSO) regression was conducted using the R package “glmnet” to feature selection, and 10-fold cross-validation was used to narrow down the candidate genes associated with OSCC prognosis. The risk score was calculated with the following formula:


$$Risk\;score={\sum_{i}^{6}\lambda_{i}\times Y}_{i}$$

where *λ* is decided by the minimum criteria and *Y* is the gene expression level.

The OSCC patients were divided into low- and high-risk groups based on the median risk score. The risk factor graph reflected differences between low- and high-risk groups in mortality outcomes. The Kaplan–Meier (K-M) survival analysis was used to assess the difference in overall survival between two groups by using the “survival” R package. The time-dependent receiver operating characteristic (ROC) curve was used to assess the value of the DEGs. The “survival,” “survminer,” and “timeROC” R packages were employed to perform ROC curve analysis.

### Gene Function Enrichment Analysis

To explore the enrichment of genes in potential biological processes and molecular functions from TCGA, the cluster “Profiler” package (version 3.18.0) of R software was carried out, including Gene Ontology (GO) function analysis and Kyoto Encyclopedia of Genes and Genomes (KEGG) pathway enrichment analysis. Visual analysis of data was performed by using “GOcircle” and “ggplot2” software packages. The adjusted p < 0.05 was considered to indicate a statistically significant difference in the charts.

To study the potential function of cuproptosis-related genes in CAFs, single-sample Gene Set Enrichment Analysis (ssGSEA) (www.gsea-msigdb.org/gsea/index.jsp) was carried out to research whether genes in the CAFs were enriched in meaningful biological functions. The annotated gene set c5.go.bp.v7.5.1.symbols.gmt was selected as the reference gene set. The Gene Set Variation Analysis (GSVA) R package (V1.26.0) was used to calculate normalized enrichment scores for gene sets. These gene sets were downloaded from MSigDB database version 6.1.

Human OSCC cells (HSC)-3 and HSC-3-M3 cell lines were used to explore the relationship between AFOC-DEGs and OSCC metastasis. These cell lines were obtained from the Japanese Collection of Research Bioresources (JCRB) Cell Bank (Osaka, Japan) (27).

### Protein–Protein Interaction Network Analysis

Protein–protein interaction (PPI) analysis of genes from TCGA was performed with the search functionality of STRING (http://string.embl.de/, version 11.0), and a network interaction matrix was built. The minimum required interaction score of 0.15 was the cutoff threshold. The results of the analysis were downloaded for establishing a visualization model by using Cytoscape (http://www.cytoscape.org/, version 3.9.1).

### Correlation Analysis Between Differentially Expressed Genes and Cells

A correlation analysis was also conducted by using the 361 patients’ gene data from TCGA. Expression data matrices were comprised of TPM expression profiles including DEGs containing genes related to cuproptosis or fibrogenesis. The correlation coefficient was determined by Spearman’s correlation analysis. The scatter plot showing the infiltration level between AFOC-DEGs and CAFs was conducted by TIMER2.0 (http://timer.cistrome.org/).

### Single-Cell Phenotype Correlation Analysis

The functional state of AFOC-DEGs in OSCC was assessed by CancerSEA. CancerSEA is the first integrative database aiming at decoding different functional states of cancer cells at a single-cell level. It depicts a cancer single-cell functional state map, containing 14 functional states (including angiogenesis, apoptosis, cell cycle, differentiation, DNA damage, DNA repair, epithelial-to-mesenchymal transition (EMT), hypoxia, inflammation, invasion, metastasis, proliferation, quiescence, and stemness) from 25 cancer types of 41,900 cancer single cells (28). Then, for the single-cell dataset, Spearman’s rank correlation test with the Benjamini–Hochberg FDR correction for multiple comparisons (correlation > 0.3 and FDR < 0.05) was used to identify significant correlations between gene expressions and functional state activities. T-distributed stochastic neighbor embedding (t-SNE) analysis was used to visualize the data in two dimensions with the “ggradar,” “ggalluvial,” “ggsci,” “cowplot,” and “ggplot2” packages.

### Isolation and Culture of Cancer-Associated Fibroblasts

Oral CAFs were referred to in a prior study (10). In detail, oral mucosa tissues were obtained from the buccal area in OSCC patients with a betel chewing history and no recent medication history. The tissues were rinsed 3 times with phosphate-buffered saline (PBS) solution supplemented with 100 units/ml of penicillin, 100 μg/ml of streptomycin, and 0.2 μg/ml of puromycin. Then the epithelial layer was removed, and the tissue block was cut into 1-mm^3^ size tissue pieces. The bottom of the culture flasks was moistened with several drops of calf serum and placed on the tissue. Dulbecco’s modified Eagle medium (DMEM) containing 10% fetal bovine serum (FBS) was added to the culture flasks. The flasks were incubated at 37°C in a 5% CO_2_ incubator, and they were turned over every 4 h. The medium was changed every 3 days. After 15 days, the tissues were digested with 0.25% trypsin for 3 min. Subsequently, an inverted microscope was used, and it was found that the cells were retracted and rounded, and the cell gaps were increased. Afterward, DMEM containing 10% FBS was added to terminate the digestion, and the cells were blown into a single-cell suspension. The CAFs were naturally purified after the 3rd generation, and the cultured 3rd to 10th generation cells were used for experiments. Subsequent passages were carried out every 8 days.

### Cell Viability Assay

The effect of arecoline on the proliferation of CAFs was determined by the cell counting kit-8 assay (CCK-8; Beyotime Biotechnology, Shanghai, China) according to the manufacturer’s instructions. Briefly, CAF cells were seeded onto 96-well cell culture plates with a density of 3,000 cells for each well and treated with 0.01, 0.1, 1, and 10 μg/ml of arecoline or Barcaldine (Aladdin, Shanghai, China) for 48 h. Meanwhile, the above operation was repeated with the groups of 0.01 μg/ml of arecoline and 1 μg/ml of arecaidine, 0.1 μg/ml of arecoline and 1 μg/ml of arecaidine, 1 μg/ml of arecoline and 0.01 μg/ml of arecaidine, 0.1 μg/ml of arecoline and 0.01 μg/ml of arecaidine, and 0.1 μg/ml of arecoline and 0.1 μg/ml of arecaidine, respectively. At the beginning time, 24 and 48 h after cultivation (0 h as the baseline), CCK-8 (10 μl) was added to the individual cell culture medium of each group for determining the optical density (OD) values. The OD values were measured at 450 nm to determine cell survival with the enzyme-labeling instrument (Elx808, BioTek, Winooski, VT, USA).

### Statistical Analysis

All statistical analyses were performed in R software (version 4.0.1; https://www.r-project.org/). Log-rank test was used to analyze the significance of the p-values ​​for hazard ratio (HR) in the model. Wilcoxon tests were used to compare the means of AFOC-DEG expression in different groups. The Box–Cox transformation and Welch’s t-test were applied for analysis of differences in cuproptosis-related gene expression after arecoline treatment. Equality of variance between groups was tested with Levene’s test. The Shapiro–Wilk normality test was used for the normality test. Spearman’s correlation coefficients were used to assess the correlation between AFOC-DEGs, marker genes, and infiltrating cells. Experimental data were analyzed with a t‐test with significance at p < 0.05. * means p < 0.05, and ** means p < 0.01.

## Result

**Figure 2:**
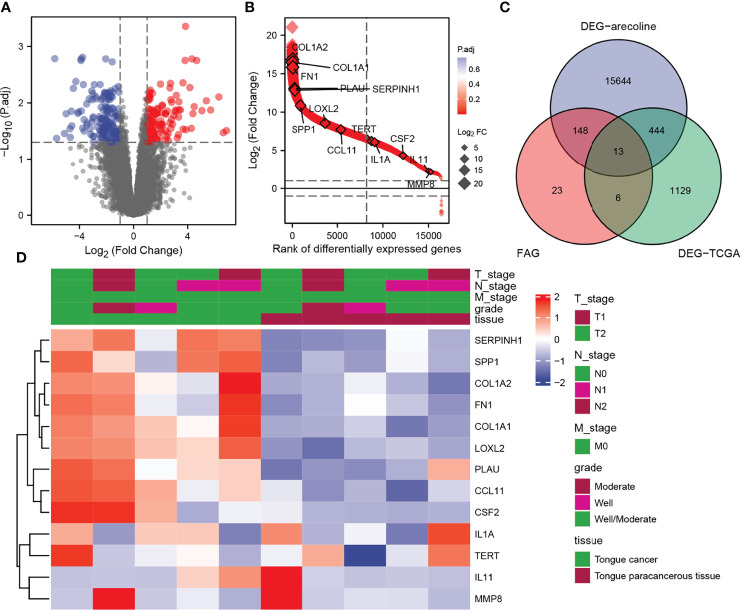
Identification of DEGs in arecoline-related OSCC. **(A)** Volcano plot shows all DEGs screened from the GEO database. **(B)** The expression levels of the thirteen DEGs screened. **(C)** Venn diagram demonstrates the intersections of genes between GEO, TCGA, and FAG databases. **(D)** Heatmap shows the association of the selected DEGs with the TNM stage and pathological stage. DEGs, differentially expressed genes; OSCC, oral squamous cell carcinoma; GEO, Gene Expression Omnibus; TNM, tumor node metastasis.

In the GSE139869 dataset, we screened for DEGs in arecoline-related OSCC, and a total of 111073 DEGs were discovered and shown on the volcano map (**Figure 2A**). The Venn diagram exhibits the intersections of the genes between the GSE139869 dataset, TCGA-OSCC dataset, and fibrosis-associated genes (FAGs; obtained from FibROAD) database (**Figure 2C**). Thirteen co-differentially expressed genes were identified, all of which were upregulated (**Figure 2B**). In addition, the association of these thirteen genes with the tumor node metastasis (TNM) stage, pathological stage, and tissue was shown in the heatmap (**Figure 2D**), which indicated that the expression level of these thirteen DEGs in cancer tissues was significantly higher than that in para-cancerous tissues. However, there was no clear correlation between these DEGs and the TNM stage or pathological stage.

### Construction of the Prognostic Gene Signature

**Figure 3:**
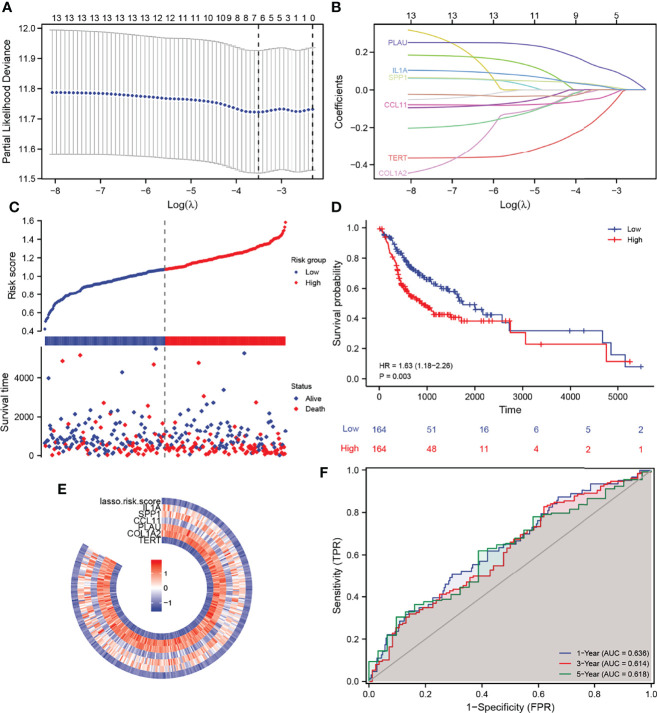
Construction of a prognostic gene signature and its predictive power. **(A)** LASSO–Cox regression for feature selection to determine optimal parameters. **(B)** AFOC-DEGs obtained by triple cross-validation of LASSO analysis. **(C)** Risk factor graph shows the distribution of the risk scores and the correlation between the risk scores and survival data. **(D)** K-M survival curve shows the difference in survival time between the high-risk group and the low-risk group. **(E)** Heatmap shows the relationship between the expression levels of AFOC-DEGs and the risk scores. **(F)** ROC shows the specificity and sensitivity of the prognostic model for predicting survival time (1, 3, and 5 years). DEGs, differentially expressed genes; LASSO, least absolute shrinkage and selection operator; K-M, Kaplan–Meier; ROC, receiver operating characteristic.

Based on the thirteen DEGs, we performed a LASSO Cox regression analysis to test the prognostic model, which showed that the model predicted best when six genes were included (**Figure 3A**). Through the regression analysis, we ascertained six AFOC-DEGs, namely, plasminogen activator urokinase (PLAU; β = 0.127), interleukin-1α (IL1A; β = 0.022), secreted phosphoprotein 1 (SPP1; β = 0.025), C-C motif chemokine ligand 11 (CCL11; β = −0.049), telomerase reverse transcriptase (TERT; β = −0.161), and collagen type I alpha 2 (COL1A2; β = −0.006) (**Figure 3B**), and the risk score was calculated using the following formula:


Risk score=0.127× [PLAU]+0.022×[IL1A]+0.025×[SPP1]−0.049×[CCL11]−0.161×[TERT]−0.006×[COL1A2]

Meanwhile, we created a heatmap to show the relationship between the expression level of these six genes and the risk scores calculated by the prognostic model (**Figure 3E**). The distribution of the risk scores and the correlation between the risk scores and survival data are revealed in **Figure 3C**, in which the patients were divided into low- and high-risk groups according to the median value of the risk scores (median value = 1.09162397). The risk factor graph demonstrated that the high-risk patients suffered higher mortality than the low-risk ones. Similar to this result, the K-M survival curve proved that the survival probability of the high-risk group decreased significantly compared with the low-risk group (**Figure 3D**, p = 0.003). Additionally, the ROC curves of the prognosis model for the survival probability at 1, 3, and 5 years are shown in **Figure 3F**. All the area under the curve (AUC) values were higher than 0.6 with the maximum AUC value reaching 0.636, certifying the satisfying specificity and sensitivity of the prognosis model. The patients’ baseline data are displayed in **Table 1**.

**Table 1:** Patients’ baseline data.

Characteristic	Low	High	p
n	164	164	
T, n (%)			0.374
T1	7 (2.2%)	11 (3.4%)	
T2	48 (14.8%)	56 (17.2%)	
T3	43 (13.2%)	39 (12%)	
T4	63 (19.4%)	51 (15.7%)	
TX	2 (0.6%)	5 (1.5%)	
N, n (%)			0.029
N0	75 (23.1%)	92 (28.3%)	
N1	29 (8.9%)	27 (8.3%)	
N2	53 (16.3%)	35 (10.8%)	
N3	3 (0.9%)	0 (0%)	
NX	3 (0.9%)	8 (2.5%)	
M, n (%)			0.016
M0	160 (49.2%)	149 (45.8%)	
M1	0 (0%)	2 (0.6%)	
MX	3 (0.9%)	11 (3.4%)	
Stage, n (%)			0.033
Stage I	7 (2.2%)	4 (1.3%)	
Stage II	61 (19.2%)	82 (25.8%)	
Stage IV	93 (29.2%)	71 (22.3%)	
Gender, n (%)			0.551
Female	48 (14.6%)	54 (16.5%)	
Male	116 (35.4%)	110 (33.5%)	
Grade, n (%)			0.028
G1	18 (5.5%)	34 (10.4%)	
G2	112 (34.4%)	87 (26.7%)	
G3	32 (9.8%)	35 (10.7%)	
G4	0 (0%)	2 (0.6%)	
GX	2 (0.6%)	4 (1.2%)	
Anatomy, n (%)			0.116
Alveolar ridge	10 (3%)	8 (2.4%)	
Base of tongue	14 (4.3%)	9 (2.7%)	
Buccal mucosa	11 (3.4%)	11 (3.4%)	
Floor of mouth	27 (8.2%)	34 (10.4%)	
Hard palate	6 (1.8%)	1 (0.3%)	
Oral cavity	28 (8.5%)	44 (13.4%)	
Oral tongue	68 (20.7%)	57 (17.4%)	

### Expression of AFOC-DEGs in Oral Squamous Cell Carcinoma Compared With Normal Tissues

**Figure 4:**
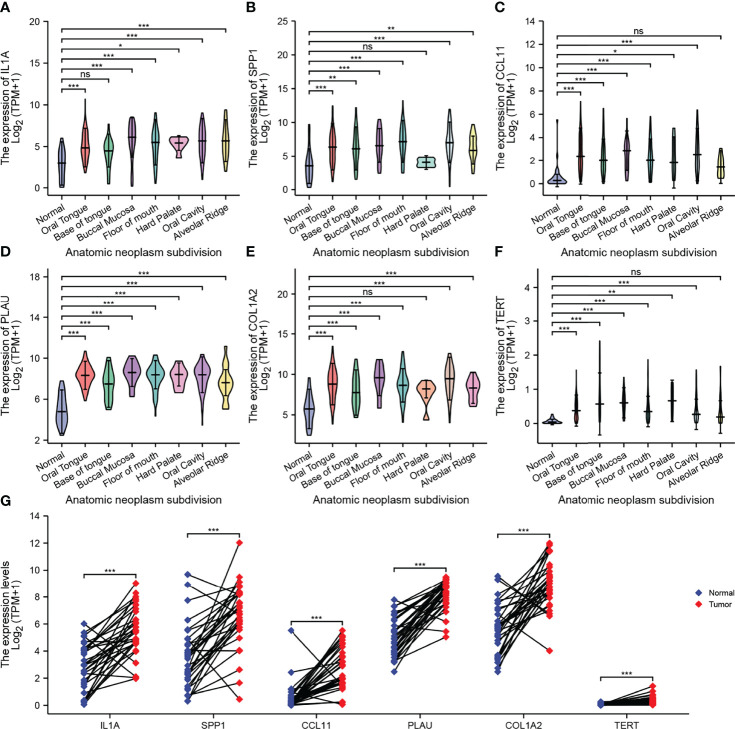
Expression of AFOC-DEGs in OSCC compared with normal tissues. **(A)** Differential expression of IL1A in OSCC of various parts of the oral cavity; a rank-sum test was used to analyze the data (n = 328, *p < 0.05; ***p < 0.001; ns, not significant). **(B)** Differential expression of SPP1 in OSCC of various parts of the oral cavity; a rank-sum test was used to analyze the data (n = 328, **p < 0.01; ***p < 0.001; ns, not significant). **(C)** Differential expression of CCL11 in OSCC of various parts of the oral cavity; a rank-sum test was used to analyze the data (n = 328, *p < 0.05; ***p < 0.001; ns, not significant). **(D)** Differential expression of PLAU in OSCC of various parts of the oral cavity; a rank-sum test was used to analyze the data (n = 328, ***p < 0.001). **(E)** Differential expression of COL1A2 in OSCC of various parts of the oral cavity; a rank-sum test was used to analyze the data (n = 328, ***p < 0.001; ns, not significant). **(F)** Differential expression of TERT in OSCC of various parts of the oral cavity; a rank-sum test was used to analyze the data (n = 328,**p < 0.01; ***p < 0.001; ns, not significant). **(G)** Differential expression of AFOC-DEGs in OSCC and para-cancer tissues; a paired rank-sum test was used to analyze the data (n = 328, ***p < 0.001). DEGs, differentially expressed genes; OSCC, oral squamous cell carcinoma.

To prove that the AFOC-DEGs screened out were indeed differentially expressed in OSCC, we compared the expression of these AFOC-DEGs in OSCC at various parts in the oral cavity with those in normal tissues (**Figures 4A–F**). Through analyses, we found that compared with normal tissues, AFOC-DEGs were highly expressed to different degrees in various regions of cancer tissues in the oral cavity, especially in the oral tongue and base of the tongue. In addition, AFOC-DEGs also showed significant expression differences in cancer tissues and para-cancer tissues, and their expression levels were significantly increased in cancer (**Figure 4G**).

### Functional Enrichment Analyses of AFOC-DEGs

**Figure 5:**
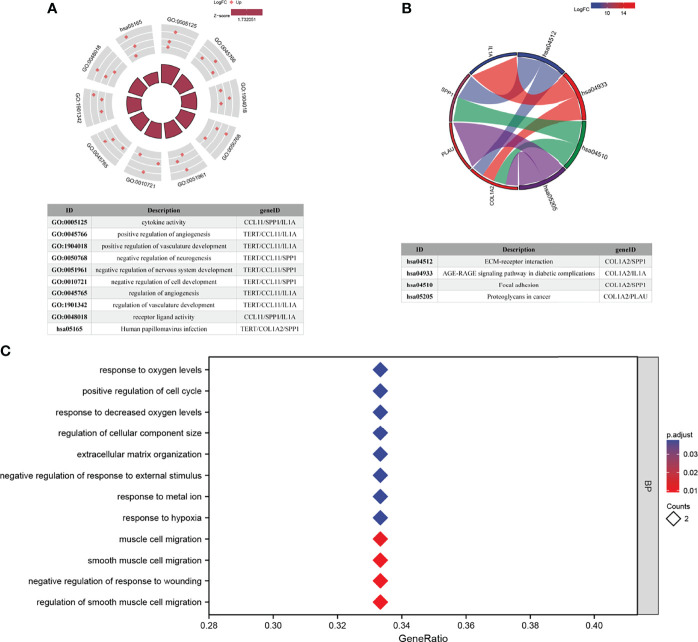
Functional enrichment analyses of AFOC-DEGs. **(A)** GO and KEGG pathway enrichment data for AFOC-DEGs (the top 10 are shown). **(B)** KEGG pathway enrichment data for AFOC-DEGs (the top 4 are shown). **(C)** GO pathway enrichment data for AFOC-DEGs (the top 12 are shown). GO, Gene Ontology; KEGG, Kyoto Encyclopedia of Genes and Genomes.

GO analysis and KEGG pathway enrichment analysis were performed to explore the potential biological functions of AFOC-DEGs (**Figures 5A–C**). A total of 49 GO terms of biological process, 1 GO term of cellular component, 3 GO terms of molecular function, and 5 KEGG pathways were identified to be significant. Among these, we discovered that the AFOC-DEGs were significantly associated with the biological processes including response to hypoxia, response to metal ions, response to decreased oxygen levels, and response to oxygen levels, which proved that the AFOC-DEGs might have an internal relationship with cuproptosis. To further confirm this conjecture, we performed ssGSEA to demonstrate the link between the biological processes, the genes associated with cuproptosis, and the TCA cycle (**Supplementary Figure 1**, **Supplementary Table 1**). The complete results of GO and KEGG analyses could be found in **Supplementary Table 2**.

### Correlation Analysis Between AFOC-DEGs and Cuproptosis-Related Genes

**Figure 6:**
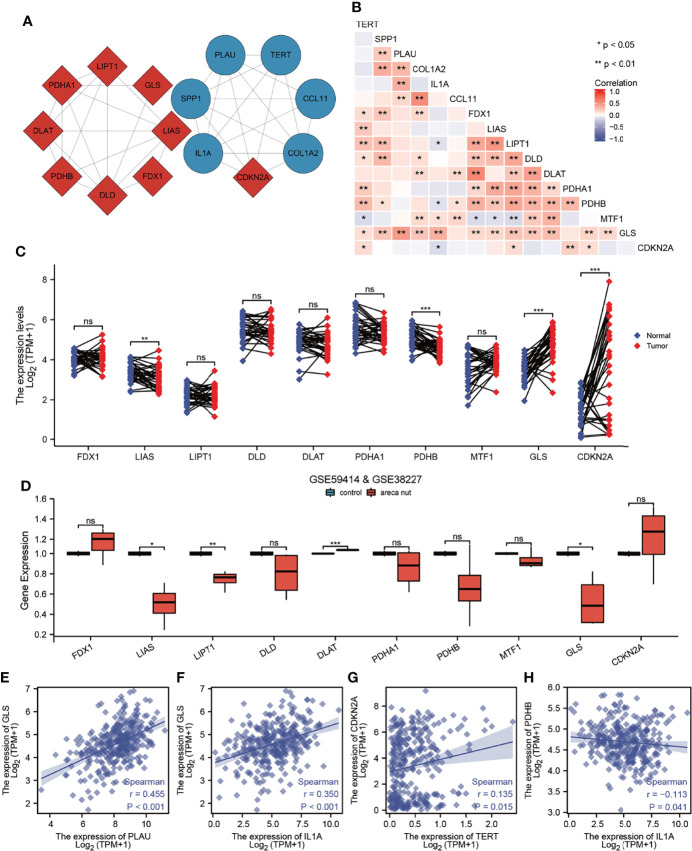
Correlation analysis between AFOC-DEGs and cuproptosis-related genes. **(A)** PPI network shows bonding between AFOC-DEGs and cuproptosis-related genes. **(B)** Heatmap shows the correlation between AFOC-DEGs and cuproptosis-related genes; Spearman’s correlation analyses were used to analyze the correlations (n = 328, *p < 0.05; **p < 0.01). **(C)** Differential expression of copper cuproptosis-related genes in OSCC and para-cancer tissues; a paired rank-sum test was used to analyze the data (n = 328, **p < 0.01; ***p < 0.001; ns, not significant). **(D)** The difference of cuproptosis-related genes’ expression in normal oral cells and oral cells treated with 5 μg/ml of areca nut water extract, Box–Cox transformation, and Welch’s t-test were used to analyze the data (n = 7, *p < 0.05; **p < 0.01; ***p < 0.001; ns, not significant). **(E)** Spearman’s correlation analysis between PLAU and GLS (n = 328, r = 0.455, p < 0.001). **(F)** Spearman’s correlation analysis between IL1A and GLS (n = 328, r = 0.350, p < 0.001). **(G)** Spearman’s correlation analysis between TERT and CDKN2A (n = 328, r = 0.135, p = 0.015). **(H)** Spearman’s correlation analysis between IL1A and PDHB (n = 328, r = −0113, p = 0.041). PPI, protein–protein interaction; OSCC, oral squamous cell carcinoma.

To further determine whether AFOC-DEGs were correlated with cuproptosis, we obtained PPI networks with the STRING tool (**Figure 6A**). Nine cuproptosis-related genes and six AFOC-DEGs were included in the network. It was found that there was a tight bonding between CDKN2A and AFOC-DEGs. A Spearman’s correlation analysis was also performed to analyze the correlation between AFOC-DEGs and cuproptosis-related genes. Through the analysis, we found that the six AFOC-DEGs were closely related to certain cuproptosis-related genes (**Figure 6B**). The highest positive correlation was between PLAU and GLS (R = 0.455; p < 0.01). Then, the second-highest positive correlation was between IL1A and GLS (R = 0.350; p < 0.01).

Furthermore, we compared the differential expressions of cuproptosis-related genes between OSCC tissues and para-cancer tissues and found that four genes (LIAS, PDHB, GLS, and CDKN2A) were differentially expressed. LIAS and PDHB were cuproptosis promoters with low expression in cancer tissues, while GLS and CDKN2A were cuproptosis suppressors with high expression in cancer tissues (**Figure 6C**). Meanwhile, decreased expressions of LIAS and LIPT1 and the increased expressions of DLAT and GLS were also found in the oral normal cells treated with 5 μg/ml of arecoline for 48 h (**Figure 6D**). Based on these results, we made scatter plots of the correlation between these four cuproptosis-related genes and AFOC-DEGs; four of them are shown in **Figures 6E–H**. All the above results confirmed the close relationship between AFOC-DEGs and cuproptosis.

### Correlation Between Arecoline-Associated Oral Squamous Cell Carcinoma and Cancer-Associated Fibroblasts

**Figure 7:**
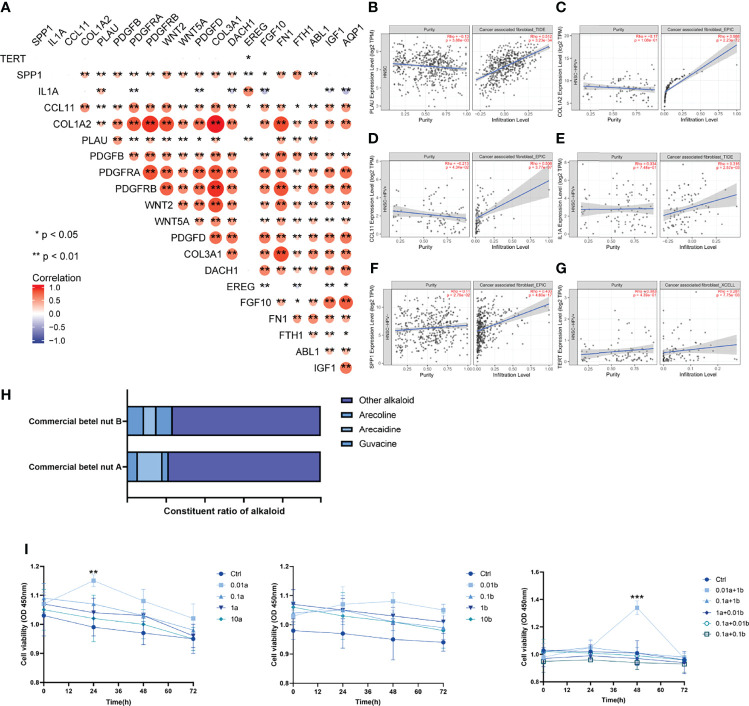
Correlation between arecoline-associated OSCC and CAFs. **(A)** Heatmap shows the correlation between AFOC-DEGs and characteristic genes associated with CAFs; Spearman’s correlation analyses were used to analyze the correlations (n = 328, *p < 0.05; **p < 0.01). **(B)** The correlation between PLAU and CAF infiltration; the correlation analysis was done by TIMER2.0 (n = 522, rho = 0.512, p < 0.001). **(C)** The correlation between COL1A2 and CAF infiltration; the correlation analysis was done by TIMER2.0 (n = 98, rho = 0.988, p < 0.001). **(D)** The correlation between CCL11 and CAF infiltration; the correlation analysis was done by TIMER2.0 (n = 98, rho = 0.508, p < 0.001). **(E)** The correlation between IL1A and CAF infiltration; the correlation analysis was done by TIMER2.0 (n = 98, rho = 0.316, p = 0.003). **(F)** The correlation between SPP1 and CAF infiltration; the correlation analysis was done by TIMER2.0 (n = 422, rho = 0.403, p < 0.001). **(G)** The correlation between TERT and CAF infiltration; the correlation analysis was done by TIMER2.0 (n = 98, rho = 0.281, p = 0.008). **(H)** Effects of arecoline stimulation on cell viability; a refers to arecoline, and b refers to arecaidine; all experiments were performed in three repetitions; a t-test was used to analyze the data (**p < 0.01; ***p < 0.001). **(I)** Commercial betel nut composition diagram shows that arecoline and arecaidine are the main alkaloids in betel nut. OSCC, oral squamous cell carcinoma; CAFs, cancer-associated fibroblasts.

Spearman’s correlation analysis was applied to analyze the correlation between AFOC-DEGs and characteristic genes associated with CAFs (**Figure 7A**), and the results showed a broad correlation among them. At the same time, immune infiltration analyses were also used to determine the correlation between AFOC-DEGs and CAF infiltration (**Figures 7B–G**), all of which demonstrated that AFOC-DEGs could affect the activity of CAFs.

To further verify the effect of arecoline on CAFs in OSCC, we stimulated CAFs with arecoline, arecaidine (another major alkaloid in betel nut), the combination of these two alkaloids, and normal saline, respectively. The results showed that the cell viability of CAFs stimulated with arecoline was significantly increased compared with the control group, and this effect was more obvious when combined with arecaidine (**Figures 7H, I**). These results directly proved that arecoline could induce CAF proliferation.

### Single-Sample Gene Set Enrichment Analysis Between Cuproptosis-Related Gene Expression and Fibroproliferation-Related Biological Processes

**Figure 8:**
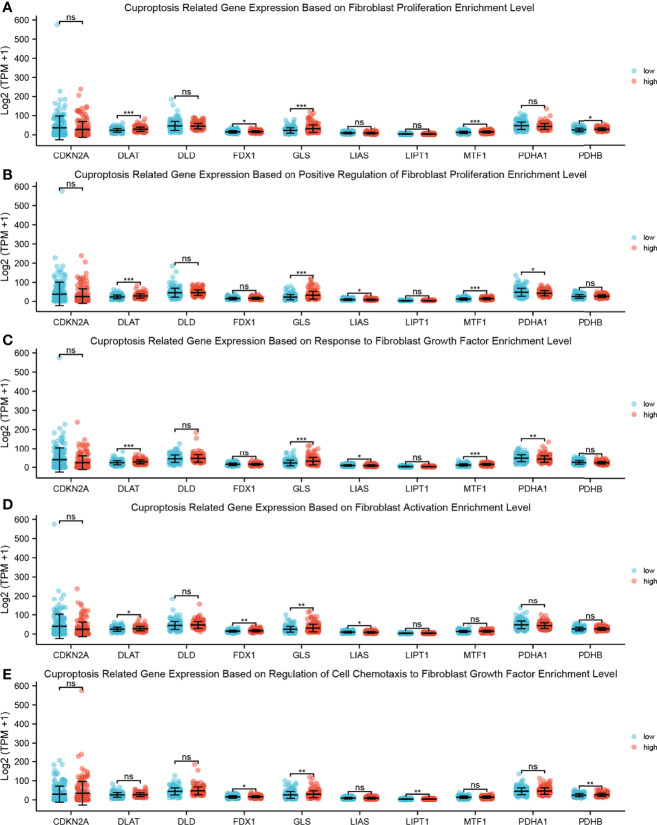
ssGSEA between cuproptosis-related gene expression and fibroproliferation-related biological processes. **(A)** Dot plot shows the relationship between cuproptosis-related gene expression and fibroblast proliferation enrichment level; a rank-sum test was used to analyze the data (n = 328, *p < 0.05; ***p < 0.001; ns, not significant). **(B)** Dot plot shows the relationship between cuproptosis-related gene expression and positive regulation of fibroblast proliferation enrichment level; a rank-sum test was used to analyze the data (n = 328, *p < 0.05; ***p < 0.001; ns, not significant). **(C)** Dot plot shows the relationship between cuproptosis-related gene expression and response to fibroblast growth factor enrichment level; a rank-sum test was used to analyze the data (n = 328, *p < 0.05; **p < 0.01; ***p < 0.001; ns, not significant). **(D)** Dot plot shows the relationship between cuproptosis-related gene expression and fibroblast activation enrichment level; a rank-sum test was used to analyze the data (n = 328, *p < 0.05; **p < 0.01; ns, not significant). **(E)** Dot plot shows the relationship between cuproptosis-related gene expression and regulation of cell chemotaxis to fibroblast growth factor enrichment level; a rank-sum test was used to analyze the data (n = 328, *p < 0.05; **p < 0.01; ns, not significant). ssGSEA, single-sample Gene Set Enrichment Analysis.

To demonstrate the relationship between CAFs and cuproptosis, we performed ssGSEAs on cuproptosis-related gene expression and fibroproliferation-related biological processes, including fibroblast proliferation, positive regulation of fibroblast proliferation, response to fibroblast growth factor, fibroblast activation, and regulation of cell chemotaxis to fibroblast growth factor (**Figures 8A–E**). The samples were divided into high-level and low-level groups according to the median value of enrichment scores, which represented the activity level of biological processes co-regulated by members of the gene set. It was found that there was a significant difference in the proliferation level of fibroblasts between the high-level and low-level groups. The expression level of cuproptosis suppressor genes GLS and MTF1 was significantly increased in the high-level group, which revealed that a tight link might exist between fibroblast proliferation and cuproptosis.

### AFOC-DEGs Were Associated With Oral Squamous Cell Carcinoma Metastasis

**Figure 9:**
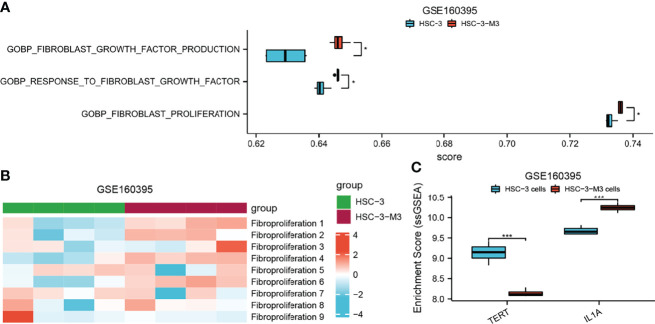
The role of AFOC-DEGs and fibroproliferation-related biological processes in the metastasis of HSC. **(A)** Heatmap shows the relationship between fibroproliferation-related biological processes and HSC metastasis. **(B)** Dot plot shows differences in fibroproliferation-related biological processes between HSCs and metastasis HSCs; a rank-sum test was used to analyze the data (n = 328, *p < 0.05). **(C)** Dot plot shows differences in the expression of TERT and LI1A between HSCs and metastasis HSCs; a rank-sum test was used to analyze the data (n = 328, ***p < 0.001).

To further explore the role of AFOC-DEGs in OSCC, we analyzed the relationship between fibroproliferation-related biological processes and tumor metastasis by ssGSEA. We found that fibroproliferation-related biological processes, especially fibroblast growth factor production, response to fibroblast growth factor, and fibroblast proliferation, were activated to varying degrees in metastatic HSCs (**Figures 9A, B**), which proved the tight association between the fibroproliferation and the metastasis of OSCC. The explanation of the fibroproliferation-related biological processes is shown in **Supplementary Table 3**. In addition, further analysis revealed that in metastatic HSCs, the expression of TERT was significantly decreased, while the expression of IL1A was significantly increased, suggesting that cuproptosis might play an essential role in tumor metastasis (**Figure 9C**).

**Figure 10:**
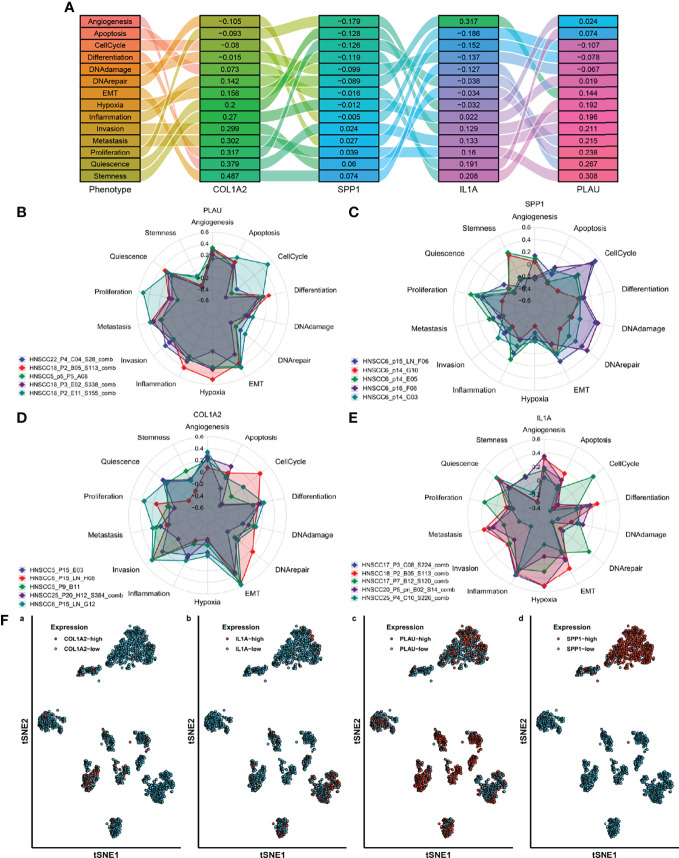
Single-cell classification functional enrichment results of AFOC-DEGs. **(A)** Phenotypic Sankey diagram of cell function enrichment. **(B)** Radar map of phenotype distribution of OSCC cells with high PLAU expression. **(C)** Radar map of phenotype distribution of OSCC cells with high SPP1 expression. **(D)** Radar map of phenotype distribution of OSCC cells with high COL1A2 expression. **(E)** Radar map of phenotype distribution of OSCC cells with high IL1A expression. **(F)** TSNE cell sorting results from single-cell sequencing. OSCC, oral squamous cell carcinoma.

Based on the above results, to determine the effect of AFOC-DEGs on the cellular phenotype of OSCC cells, t-SNE was applied to cell-sort the single-cell sequencing results of 2073 OSCC cells in the GSE84756 dataset, thereby identifying cells with high expression of AFOC-DEGs in OSCC (**Figure 10F**). COL1A2, IL1A, PLAU, and SPP1 were shown to have cellular significance. By enriching these cells with high AFOC-DEG expression and cell phenotype, we found that they were highly correlated with EMT, invasion, metastasis, etc. (**Figures 9B–E**). The correlations with EMT generally exceeded 0.3, and the correlation between cells with high COL1A2 expression and EMT reached 0.6. These results uncovered that AFOC-DEGs were highly related to the metastasis of OSCC cells (**Figure 9A**).

## Discussion

Chewing betel nut has been verified as a potent carcinogenic factor for OSCC. The primary active ingredients of betel nut are the areca alkaloids, especially arecoline, leading to fibroblast proliferation and fibrotic changes (29). The genotoxicity of arecoline inducing chromosomal damage and gene mutations may account for the pathogenesis, including a DNA damage response cascade involving phosphorylation of ataxia-telangiectasia (ATM) kinase and its downstream targets checkpoint kinase 1/2 (Chk1/2), p53, and Nbs1, leading to a G2/M cell cycle arrest (7, 30). Apart from these alternations, protein expression of several other cell cycle regulatory molecules like cdc25c in basal carcinoma cells, cyclin B1 and Wee-1 in KB epithelial cells, and cyclin D1, cyclin A, cyclin E, CDK4, and CDK2 in HaCaT keratinocytes are modulated by arecoline. However, the detailed mechanism for the specific molecules of arecoline in OSCC has not been fully clarified. In this study, we acquired arecoline-related OSCC samples from TCGA-OSCC dataset and GSE139869 gene array and identified six AFOC-DEGs with a highly reliable prediction model for OSCC. Expression of AFOC-DEGs in OSCC compared with normal tissues also emerged as an increased result. The functional enrichment and correlation analysis verified the potential effect of cuproptosis in arecoline-related OSCC *via* AFOC-DEGs. The correlation between AFOC-DEGs, cuproptosis, and CAFs was also illustrated by correlation analyses. The immune infiltration analysis was also applied. The cell viability assessment demonstrated the stimulative influence of arecoline on CAFs in OSCC. The single-cell analysis of OSCC also revealed marked relevance between AFOC-DEGs and EMT, invasion, and metastasis. These discoveries offered a novel perspective into cuproptosis, CAFs, and prognosis of patients with arecoline-related OSCC *via* the investigation of AFOC-DEGs.

Several researchers reported the survival analysis of head and neck squamous cell carcinoma (HNSCC) cohorts with betel nut exposure and associated prognosis factors. It was found that the expression of DNA repair genes ATM and BRCA1 was suppressed in betel nut-associated HNSCC due to arecoline. Low expression of either ATM or BRCA1 was related to poor overall survival and presented as an independent prognostic factor (31). Here, for the first time, we screened thirteen DEGs based on arecoline-related OSCC samples from the GSE139869 gene array and TCGA-OSCC dataset. With LASSO regression analysis, we established a novel prognosis model for arecoline-related OSCC patients depending on six AFCO-DEGs: PLAU, IL1A, SPP1, CCL11, TERT, and COL1A2. The K-M curve demonstrated that the patients with high-risk scores in this model suffered a poorer prognosis as compared with the low-risk groups. The ROC curves for the survival probability at 1, 3, and 5 years also revealed great specificity and sensitivity of this prognosis model with all the AUC values higher than 0.6. The comparison of the AFOC-DEG expression in cancer tissues from different parts of the oral cavity and the corresponding normal tissues further confirmed that AEOC-DEGs were upregulated in various kinds of OSCC.

With t-SNE, we analyzed the single-cell sequencing results of 2,073 OSCC cells identifying the ones with high expression of AFOC-DEGs. By enrichment analyses, COL1A2, IL1A, PLAU, and SPP1 were discovered to be highly correlated with EMT, invasion, and metastasis with cellular significance. COL1A2 is a subtype of type I collagen produced by stromal fibroblasts and cancer cells, and previous researchers have identified it as a potential biomarker correlated with immune infiltration in the esophageal carcinoma TME (32). The high expression of IL-1α has been regarded as a tumor aggressiveness promoter leading to poor survival of HNSCC patients (33, 34). Encoding a secreted serine protease urokinase, PLAU has been attested to be associated with poor prognosis in HNSCC, the potential mechanism of which may be the dysfunction of the PI3K-Akt pathway and EMT process or aberrant proportions of immune cells (35, 36). SPP1 is an integrin-binding glycol-phosphoprotein whose overexpression exerts diverse tumor-associated functions such as proliferation, invasion, migration, angiogenesis, and metastasis. The present pan-cancer analyses revealed that SPP1 and its correlated genes might perform their efficacy by regulating tumor immune infiltration in various malignant tumors including HNSCC (37, 38). The reactivated and overexpressed TERT increases the telomerase activity resulting in abnormal cell proliferation and even replicative immortality. The enhanced TERT promoter activity was also demonstrated to be related to betel chewing, tumorigenesis, tumor development, and poor prognosis in OSCC by the previous prevalence studies (39, 40). CCL11 signaling regulates the TME impacting cancer progression; however, its specific mechanism of action for OSCC or other kinds of HNSCC has not been reported. Except for TERT, we first reported the relationship of other AFOC-DEGs with arecoline-related OSCC and the potential mechanisms.

Based on the gene function enrichment results of AFOC-DEGs in response to metal ion and oxygen levels, we further investigated the novel molecular mechanism of AFOC-DEGs in the formation and development of OSCC. Copper is involved in tumorous development and spreading *via* accelerating various biological functions including EMT, angiogenesis, cell proliferation, and metabolism transformation (24, 41). A recent study found an undiscovered regulated cell death mediated by copper, the appearance of which relied on mitochondrial respiration regulation *via* protein lipoylation in the TCA cycle (42). Among the genes engaged in the cuproptosis, FDX1, LIAS, LIPT1, DLD, DLAT, PDHA1, and PDHB were confirmed to be positive regulators, and MTF1, GLS, and CDKN2A were considered negative ones. In the PPI network, it was discovered that CDKN2A had tight bonding sites with AFOC-DEGs. Spearman’s correlation analysis exhibited a statistically significant correlation between AFOC-DEGs and cuproptosis-relevant genes especially LIAS, PDHB, GLS, and CDKN2A. The differential expression analyses of OSCC tissues and para-cancer tissues demonstrated that LIAS and PDHB were suppressed in cancer tissues, but GLS and CDKN2A were accelerated. Hence, we speculated that AFOC-DEGs inhibited cuproptosis leading to the downstream effects in OSCC.

CAFs have been regarded as the most prominent non-immune tumor-promoting cells within cancers, especially in HNSCC (43). In summary, CAFs exert manifold pro-tumoral functions in oral cancers *via* immunosuppression, metabolic switch, tumor proliferation, angiogenesis, tumor invasion, and therapy resistance (44–49). In a clinical study of OSCC patients, the scholars proved that lymph node metastasis occurred more frequently in the CAF-positive group, and the survival rate was significantly poorer in this group (15). However, treatments designed to target CAFs have not been successfully applied in clinical practice, which may due to a limited understanding of the CAFs’ molecular and functional phenotypes in specific tumors. In the previous study, we proved that arecoline increased the PDE4A activity inhibiting the cAMP/Epac1 pathway in TGF-β-activated buccal mucosal fibroblasts, which ultimately facilitated OSF, a kind of precancerous lesion in OSCC (10). In this research, we displayed a broad correlation among AFOC-DEGs and other CAF genes with Spearman’s analysis. The immune infiltration analysis also revealed a high positive relationship between CAFs and AFOC-DEGs, especially COL1A2, PLAU, and CCL11. The CAFs from OSCC stimulated with arecoline also showed higher cell viability as compared with the control group, directly certifying the promoting impact of arecoline on CAFs in OSCC. PLAU was found to promote esophageal squamous cell carcinoma progression by the conversion of fibroblasts into inflammatory CAFs *via* uPAR/Akt/NF-κB/IL8 pathway (50). What is more, other AFOC-DEGs were first demonstrated to be promoters for CAFs in OSCC, which might be potential targets for future therapy.

Previous studies have demonstrated the effect of copper in inducing CAF apoptosis through ROS/MAPK and ferroptosis pathways. However, in CAFs, the direct impact of cuproptosis has not been studied yet (51). In the ssGSEAs of cuproptosis-related gene expression and fibroproliferation-related biological processes, we found that cuproptosis suppressor genes GLS and MTF1 were significantly increased in the high level of fibroproliferation-related biological processes, indicating their latent correlative mechanism.

There are still ineluctable limitations in our research. Though we have screened for AFOC-DEGs with bioinformatics analysis attempting to interpret the roles of cuproptosis and CAFs in arecoline-related OSCC, more *in vitro* and *in vivo* experiments are needed for direct biological evidence to demonstrate the exact mechanism of action. Meanwhile, the interactive effect of cuproptosis and CAFs requires more exploration to expound. In further research, we plan to knock down or overexpress AFOC-DEGs in CAFs and explore their variation in cell migration, invasion, and proliferation and potential upstream or downstream influence *in vitro* and *in vivo*.

## Conclusion

In conclusion, we identified six AFOC-DEGs in arecoline-related OSCC. The potential effect of cuproptosis on arecoline-related OSCC and its correlation with AFOC-DEGs were also verified. Correlation analysis also demonstrated the relationship between AFOC-DEGs, cuproptosis, and CAFs. Immune infiltration and *in vivo* experiments demonstrated a stimulatory effect of arecoline on CAFs in OSCC. Single-cell analysis of OSCC also revealed that high expression of AFOC-DEGs was closely related to OSCC metastasis. Through our study of AFOC-DEGs, we have demonstrated for the first time a close link between arecoline, cuproptosis, CAFs, and OSCC metastasis, providing a new perspective on arecoline-related OSCC. These findings suggest that copper ionophore therapy directed toward OSCC with such a pathological characteristic may be a possible treatment for improving these specific patient outcomes. In addition, CAF targeted therapy will also have a positive impact on the long-term prognosis of OSCC patients. In the future, more in-depth substantive research on arecoline, cuproptosis, and CAFs should therefore be considered.

## Data Availability Statement

Publicly available datasets were analyzed in this study. The datasets [GSE139869, GSE160395, GSE59414 and GSE38227] for this study can be found in the [GEO Database] [www.ncbi.nlm.nih.gov/geo]. The datasets [TCGA-OSCC] for this study can be found in the [TCGA Database] [https://tcga-data.nci.nih.gov/tcga]. The datasets [FAG] for this study can be found in the [FibROAD Database][https://www.fibroad.org/download.php].

## Ethics Statement

The studies involving human participants were reviewed and approved by the ethic committee of the Third Xiangya Hospital, Central South University. The patients/participants provided their written informed consent to participate in this study.

## Author Contributions

JL, SC, and YL contributed to the conception/design of the work, the collection and analysis of data, and the writing and editing of the article. The remaining authors provided editing and writing assistance. All authors contributed to the article and approved the submitted version.

## Funding

This work was supported by the Hunan Science and Health ;Join Project, 2020JJ8019.

## Conflict of Interest

The authors declare that the research was conducted in the absence of any commercial or financial relationships that could be construed as a potential conflict of interest.

## Publisher’s Note

All claims expressed in this article are solely those of the authors and do not necessarily represent those of their affiliated organizations, or those of the publisher, the editors and the reviewers. Any product that may be evaluated in this article, or claim that may be made by its manufacturer, is not guaranteed or endorsed by the publisher.

## Supplementary Material

Supplementary Figure 1 The relationship between cuproptosis and the TCA cycle The datasets [GSE139869, GSE160395, GSE59414 and GSE38227] for this study **(A)** Heat map showed the relationship between 8 copper-related biological processes and FAG, TNM stage, stage, gender, grade, anatomy, and TCA cycle activation level. **(B)** Violin plot showed the differences in copper-related biological processes in OSCC with high and low levels of TCA cycle, a rank-sum test was used to analyze the data (n = 328, *P < 0.05; **P < 0.01; ns, not significant). **(C)** Violin plot showed the differences in cuproptosis-related genes in OSCC with high and low levels of TCA cycle, a rank-sum test was used to analyze the data (n = 328, **P < 0.01; ***P < 0.001).

Supplementary Table 1 The explanation of the biological process of copper ion.

Supplementary Table 2 The complete results of GO and KEGG analyses.

## References

[1] Neville BW Day TA. Oral Cancer and Precancerous Lesions. CA Cancer J Clin (2002) 52(4):195–215. doi: 10.3322/canjclin.52.4.195 12139232

[2] Sung H Ferlay J Siegel RL Laversanne M Soerjomataram I Jemal A. Global Cancer Statistics 2020: GLOBOCAN Estimates of Incidence and Mortality Worldwide for 36 Cancers in 185 Countries. CA Cancer J Clin (2021) 71(3):209–49. doi: 10.3322/caac.21660 33538338

[3] Kaur J Srivastava R Borse V. Recent Advances in Point-of-Care Diagnostics for Oral Cancer. Biosens Bioelectron (2021) 178:112995. doi: 10.1016/j.bios.2021.112995 33515983

[4] Zeng H Chen W Zheng R Zhang S Ji JS Zou X. Changing Cancer Survival in China During 2003-15: A Pooled Analysis of 17 Population-Based Cancer Registries. Lancet Glob Health (2018) 6(5):e555–67. doi: 10.1016/S2214-109X(18)30127-X 29653628

[5] Koyfman SA Ismaila N Crook D D'Cruz A Rodriguez CP Sher DJ. Management of the Neck in Squamous Cell Carcinoma of the Oral Cavity and Oropharynx: ASCO Clinical Practice Guideline. J Clin Oncol (2019) 37(20):1753–74. doi: 10.1200/JCO.18.01921 PMC7098829 30811281

[6] Trivedy C Baldwin D Warnakulasuriya S Johnson N Peters T. Copper Content in Areca Catechu (Betel Nut) Products and Oral Submucous Fibrosis. Lancet (1997) 349(9063):1447. doi: 10.1016/S0140-6736(97)24020-1 9164320

[7] group IMV. Carcinogenicity of Acrolein, Crotonaldehyde, and Arecoline. Lancet Oncol (2021) 22(1):19–20. doi: 10.1016/S1470-2045(20)30727-0 33248467

[8] Personal Habits and Indoor Combustions. Volume 100 E. A Review of Human Carcinogens. IARC Monogr Eval Carcinog Risk Hum (2012) 100(Pt E):1–538. Humans IWGotEoCRt. PMC4781577 23193840

[9] Tsai YS Lee KW Huang JL Liu YS Juo SH Kuo WR. Arecoline, a Major Alkaloid of Areca Nut, Inhibits P53, Represses DNA Repair, and Triggers DNA Damage Response in Human Epithelial Cells. Toxicology (2008) 249(2-3):230–7. doi: 10.1016/j.tox.2008.05.007 18585839

[10] Zhang B Gao L Shao C Deng M Chen L. Arecoline Enhances Phosphodiesterase 4a Activity to Promote Transforming Growth Factor-Beta-Induced Buccal Mucosal Fibroblast Activation *via* cAMP-Epac1 Signaling Pathway. Front Pharmacol (2021) 12:722040. doi: 10.3389/fphar.2021.722040 34819854 PMC8606562

[11] Tlsty TD Coussens LM. Tumor Stroma and Regulation of Cancer Development. Annu Rev Pathol (2006) 1:119–50. doi: 10.1146/annurev.pathol.1.110304.100224 18039110

[12] Fiori ME Di Franco S Villanova L Bianca P Stassi G De Maria R. Cancer-Associated Fibroblasts as Abettors of Tumor Progression at the Crossroads of EMT and Therapy Resistance. Mol Canc (2019) 18(1):70. doi: 10.1186/s12943-019-0994-2 PMC6441236 30927908

[13] Park D Sahai E Rullan A. SnapShot: Cancer-Associated Fibroblasts. Cell (2020) 181(2):486–486.e1. doi: 10.1016/j.cell.2020.03.013 32302576

[14] Wu MH Hong HC Hong TM Chiang WF Jin YT Chen YL. Targeting Galectin-1 in Carcinoma-Associated Fibroblasts Inhibits Oral Squamous Cell Carcinoma Metastasis by Downregulating MCP-1/CCL2 Expression. Clin Cancer Res (2011) 17(6):1306–16. doi: 10.1158/1078-0432.CCR-10-1824 21385934

[15] Kawashiri S Tanaka A Noguchi N Hase T Nakaya H Ohara T. Significance of Stromal Desmoplasia and Myofibroblast Appearance at the Invasive Front in Squamous Cell Carcinoma of the Oral Cavity. Head Neck-J Sci Spec (2009) 31(10):1346–53. doi: 10.1002/hed.21097 19373786

[16] Teng F Tian WY Wang YM Zhang YF Guo F Zhao J. Cancer-Associated Fibroblasts Promote the Progression of Endometrial Cancer *via* the SDF-1/CXCR4 Axis. J Hematol Oncol (2016) 9:8. doi: 10.1186/s13045-015-0231-4 26851944 PMC4744391

[17] Tang S Yang L Tang X Liu M. The Role of Oxidized ATM in the Regulation of Oxidative Stress-Induced Energy Metabolism Reprogramming of CAFs. Cancer Lett (2014) 353(2):133–44. doi: 10.1016/j.canlet.2014.07.028 25069040

[18] Curtis M Kenny HA Ashcroft B Mukherjee A Johnson A Zhang Y. Fibroblasts Mobilize Tumor Cell Glycogen to Promote Proliferation and Metastasis. Cell Metab (2019) 29(1):141–55.e9. doi: 10.1016/j.cmet.2018.08.007 30174305 PMC6326875

[19] Yang J Shi X Yang M Luo J Gao Q Wang X. Glycolysis Reprogramming in Cancer-Associated Fibroblasts Promotes the Growth of Oral Cancer Through the lncRNA H19/miR-675-5p/PFKFB3 Signaling Pathway. Int J Oral Sci (2021) 13(1):12. doi: 10.1038/s41368-021-00115-7 33762576 PMC7991655

[20] Cheng HL Chang WT Hu YC Hsieh BS Huang TC Chong IW. Arecoline Increases Glycolysis and Modulates pH Regulator Expression in HA22T/VGH Hepatoma Cells, Leading to Increase of Intracellular Ca(2+), Reactive Oxygen Species, and Anoikis. J Canc (2017) 8(16):3173–82. doi: 10.7150/jca.20523 PMC5665033 29158789

[21] Ren H He G Lu Z He Q Li S Huang Z. Arecoline Induces Epithelial-Mesenchymal Transformation and Promotes Metastasis of Oral Cancer by SAA1 Expression. Cancer Sci (2021) 112(6):2173–84. doi: 10.1111/cas.14866 PMC8177782 33626219

[22] Li WD Zang CJ Yin S Shen W Sun QY Zhao M. Metformin Protects Against Mouse Oocyte Apoptosis Defects Induced by Arecoline. Cell Prolif (2020) 53(7):e12809. doi: 10.1111/cpr.12809 32557964 PMC7377942

[23] Ruiz LM Libedinsky A Elorza AA. Role of Copper on Mitochondrial Function and Metabolism. Front Mol Biosci (2021) 8:711227. doi: 10.3389/fmolb.2021.711227 34504870 PMC8421569

[24] Ge EJ Bush AI Casini A Cobine PA Cross JR DeNicola GM. Connecting Copper and Cancer: From Transition Metal Signalling to Metalloplasia. Nat Rev Canc (2022) 22(2):102–13. doi: 10.1038/s41568-021-00417-2 PMC8810673 34764459

[25] Barrett T Wilhite SE Ledoux P Evangelista C Kim IF Tomashevsky M. NCBI GEO: Archive for Functional Genomics Data Sets–Update. Nucleic Acids Res (2013) 41(Database issue):D991–5. doi: 10.1093/nar/gks1193 PMC3531084 23193258

[26] Tomczak K Czerwinska P Wiznerowicz M. The Cancer Genome Atlas (TCGA): An Immeasurable Source of Knowledge. Contemp Oncol (Pozn) (2015) 19(1A):A68–77. doi: 10.5114/wo.2014.47136 PMC4322527 25691825

[27] Ideta Y Tagawa T Hayashi Y Baba J Takahashi K Mitsudo K. Transcriptomic Profiling Predicts Multiple Pathways and Molecules Associated With the Metastatic Phenotype of Oral Cancer Cells. Cancer Genomics Proteomic (2021) 18(1):17–27. doi: 10.21873/cgp.20238 PMC7796819 33419893

[28] Yuan H Yan M Zhang G Liu W Deng C Liao G. CancerSEA: A Cancer Single-Cell State Atlas. Nucleic Acids Res (2019) 47(D1):D900–8. doi: 10.1093/nar/gky939 PMC6324047 30329142

[29] Wollina U Verma SB Ali FM Patil K. Oral Submucous Fibrosis: An Update. Clin Cosmet Investig Dermatol (2015) 8:193–204. doi: 10.2147/CCID.S80576 PMC4401336 25914554

[30] Oliveira NG Ramos DL Dinis-Oliveira RJ. Genetic Toxicology and Toxicokinetics of Arecoline and Related Areca Nut Compounds: An Updated Review. Arch Toxicol (2021) 95(2):375–93. doi: 10.1007/s00204-020-02926-9 33097969

[31] Wang YC Lee KW Tsai YS Lu HH Chen SY Hsieh HY. Downregulation of ATM and BRCA1 Predicts Poor Outcome in Head and Neck Cancer: Implications for ATM-Targeted Therapy. J Pers Med (2021) 11(5):389. doi: 10.3390/jpm11050389 34068585 PMC8151497

[32] Wang Z Chen M Qiu Y Yang Y Huang Y Li X. Identification of Potential Biomarkers Associated With Immune Infiltration in the Esophageal Carcinoma Tumor Microenvironment. Biosci Rep (2021) 41(2):BSR20202439. doi: 10.1042/BSR20202439 33543230 PMC7890403

[33] Khan IN Gibson-Corley KN Coppock JD Simons AL. Comparison of Interleukin-1 Ligand Expression by Human Papilloma Virus Status in HNSCCs. Head Neck Pathol (2022). doi: 10.1007/s12105-022-01440-x PMC9424424 35334093

[34] Leon X Bothe C Garcia J Parreno M Alcolea S Quer M. Expression of IL-1alpha Correlates With Distant Metastasis in Patients With Head and Neck Squamous Cell Carcinoma. Oncotarget (2015) 6(35):37398–409. doi: 10.18632/oncotarget.6054 PMC4741937 26460957

[35] Chen G Sun J Xie M Yu S Tang Q Chen L. PLAU Promotes Cell Proliferation and Epithelial-Mesenchymal Transition in Head and Neck Squamous Cell Carcinoma. Front Genet (2021) 12:651882. doi: 10.3389/fgene.2021.651882 34093649 PMC8173099

[36] Li Z Chen C Wang J Wei M Liu G Qin Y. Overexpressed PLAU and its Potential Prognostic Value in Head and Neck Squamous Cell Carcinoma. PeerJ (2021) 9:e10746. doi: 10.7717/peerj.10746 33520474 PMC7812932

[37] Wei T Bi G Bian Y Ruan S Yuan G Xie H. The Significance of Secreted Phosphoprotein 1 in Multiple Human Cancers. Front Mol Biosci (2020) 7:565383. doi: 10.3389/fmolb.2020.565383 33324676 PMC7724571

[38] Zeng P Zhang X Xiang T Ling Z Lin C Diao H. Secreted Phosphoprotein 1 as a Potential Prognostic and Immunotherapy Biomarker in Multiple Human Cancers. Bioengineered (2022) 13(2):3221–39. doi: 10.1080/21655979.2021.2020391 PMC8973783 35067176

[39] Arantes L Cruvinel-Carloni A de Carvalho AC Sorroche BP Carvalho AL Scapulatempo-Neto C. TERT Promoter Mutation C228T Increases Risk for Tumor Recurrence and Death in Head and Neck Cancer Patients. Front Oncol (2020) 10:1275. doi: 10.3389/fonc.2020.01275 32850388 PMC7399085

[40] Chang KP Wang CI Pickering CR Huang Y Tsai CN Tsang NM. Prevalence of Promoter Mutations in the TERT Gene in Oral Cavity Squamous Cell Carcinoma. Head Neck (2017) 39(6):1131–7. doi: 10.1002/hed.24728 28230921

[41] da Silva DA De Luca A Squitti R Rongioletti M Rossi L Machado CML. Copper in Tumors and the Use of Copper-Based Compounds in Cancer Treatment. J Inorg Biochem (2022) 226:111634. doi: 10.1016/j.jinorgbio.2021.111634 34740035

[42] Tsvetkov P Coy S Petrova B Dreishpoon M Verma A Abdusamad M. Copper Induces Cell Death by Targeting Lipoylated TCA Cycle Proteins. Science (2022) 375(6586):1254–61. doi: 10.1126/science.abf0529 PMC9273333 35298263

[43] Bienkowska KJ Hanley CJ Thomas GJ. Cancer-Associated Fibroblasts in Oral Cancer: A Current Perspective on Function and Potential for Therapeutic Targeting. Front Oral Health (2021) 2:686337. doi: 10.3389/froh.2021.686337 35048030 PMC8757746

[44] Zhang D Song Y Li D Liu X Pan Y Ding L. Cancer-Associated Fibroblasts Promote Tumor Progression by lncRNA-Mediated RUNX2/GDF10 Signaling in Oral Squamous Cell Carcinoma. Mol Oncol (2022) 16(3):780–94. doi: 10.1002/1878-0261.12935 PMC8807363 33657265

[45] Magan M Wiechec E Roberg K. CAFs Affect the Proliferation and Treatment Response of Head and Neck Cancer Spheroids During Co-Culturing in a Unique *In Vitro* Model. Cancer Cell Int (2020) 20(1):599. doi: 10.1186/s12935-020-01718-6 33353547 PMC7756959

[46] Domogauer JD de Toledo SM Howell RW Azzam EI. Acquired Radioresistance in Cancer Associated Fibroblasts is Concomitant With Enhanced Antioxidant Potential and DNA Repair Capacity. Cell Commun Signal (2021) 19(1):30. doi: 10.1186/s12964-021-00711-4 33637118 PMC7912493

[47] De Palma M Biziato D Petrova TV. Microenvironmental Regulation of Tumour Angiogenesis. Nat Rev Canc (2017) 17(8):457–74. doi: 10.1038/nrc.2017.51 28706266

[48] Monteran L Erez N. The Dark Side of Fibroblasts: Cancer-Associated Fibroblasts as Mediators of Immunosuppression in the Tumor Microenvironment. Front Immunol (2019) 10:1835. doi: 10.3389/fimmu.2019.01835 31428105 PMC6688105

[49] Kumar D New J Vishwakarma V Joshi R Enders J Lin F. Cancer-Associated Fibroblasts Drive Glycolysis in a Targetable Signaling Loop Implicated in Head and Neck Squamous Cell Carcinoma Progression. Cancer Res (2018) 78(14):3769–82. doi: 10.1158/0008-5472.CAN-17-1076 PMC6050074 29769197

[50] Fang L Che Y Zhang C Huang J Lei Y Lu Z. PLAU Directs Conversion of Fibroblasts to Inflammatory Cancer-Associated Fibroblasts, Promoting Esophageal Squamous Cell Carcinoma Progression *via* uPAR/Akt/NF-Kappab/IL8 Pathway. Cell Death Discov (2021) 7(1):32. doi: 10.1038/s41420-021-00410-6 33574243 PMC7878926

[51] Li Y Chen F Chen J Chan S He Y Liu W. Disulfiram/Copper Induces Antitumor Activity Against Both Nasopharyngeal Cancer Cells and Cancer-Associated Fibroblasts Through ROS/MAPK and Ferroptosis Pathways. Cancers (Basel) (2020) 12(1). doi: 10.3390/cancers12010138 PMC7017005 31935835

