# Supplementary figures and images for: Arecoline Is Associated With Inhibition of Cuproptosis and Proliferation of Cancer-Associated Fibroblasts in Oral Squamous Cell Carcinoma: A Potential Mechanism for Tumor Metastasis
Source: Front Oncol. 2022 Jul 7;12:925743. doi: 10.3389/fonc.2022.925743 (PMC9303015; doi:10.3389/fonc.2022.925743)

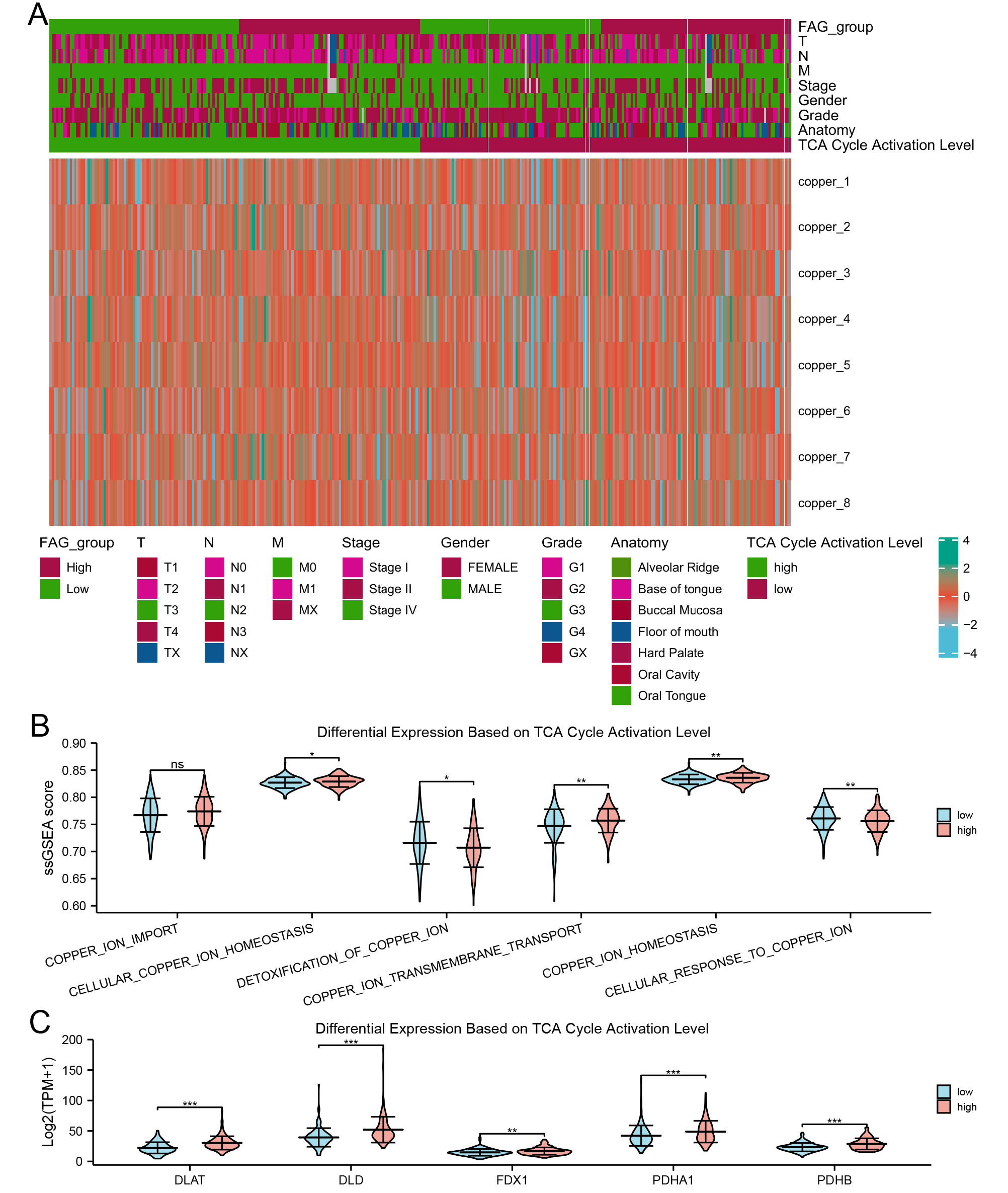

Supplement: Supplementary Figure 1 — The relationship between cuproptosis and the TCA cycle The datasets [GSE139869, GSE160395, GSE59414 and GSE38227] for this study (A) Heat map showed the relationship between 8 copper-related biological processes and FAG, TNM stage, stage, gender, grade, anatomy, and TCA cycle activation level. (B) Violin plot showed the differences in copper-related biological processes in OSCC with high and low levels of TCA cycle, a rank-sum test was used to analyze the data (n = 328, *P < 0.05; **P < 0.01; ns, not significant). (C) Violin plot showed the differences in cuproptosis-related genes in OSCC with high and low levels of TCA cycle, a rank-sum test was used to analyze the data (n = 328, **P < 0.01; ***P < 0.001). [file Image_1.tif]
