# Supplementary material for: Arecoline Is Associated With Inhibition of Cuproptosis and Proliferation of Cancer-Associated Fibroblasts in Oral Squamous Cell Carcinoma: A Potential Mechanism for Tumor Metastasis
Source: Front Oncol. 2022 Jul 7;12:925743. doi: 10.3389/fonc.2022.925743 (PMC9303015; doi:10.3389/fonc.2022.925743)
Supplement: Supplementary Table 1 — The explanation of the biological process of copper ion. [file Table_1.docx]

| GO_ID | Simplified_ID |
| --- | --- |
| GOBP_COPPER_ION_TRANSPORT | copper_1 |
| GOBP_CELLULAR_COPPER_ION_HOMEOSTASIS | copper_2 |
| GOBP_DETOXIFICATION_OF_COPPER_ION | copper_3 |
| GOBP_COPPER_ION_IMPORT | copper_4 |
| GOBP_COPPER_ION_TRANSMEMBRANE_TRANSPORT | copper_5 |
| GOBP_RESPONSE_TO_COPPER_ION | copper_6 |
| GOBP_COPPER_ION_HOMEOSTASIS | copper_7 |
| GOBP_CELLULAR_RESPONSE_TO_COPPER_ION | copper_8 |
