# Supplementary material for: Arecoline Is Associated With Inhibition of Cuproptosis and Proliferation of Cancer-Associated Fibroblasts in Oral Squamous Cell Carcinoma: A Potential Mechanism for Tumor Metastasis
Source: Front Oncol. 2022 Jul 7;12:925743. doi: 10.3389/fonc.2022.925743 (PMC9303015; doi:10.3389/fonc.2022.925743)
Supplement: Supplementary Table 2 — The complete results of GO and KEGG analyses. [file Table_2.docx]

| ONTOLOGY | ID | Description | GeneRatio | BgRatio | pvalue | p.adjust | qvalue | geneID | Count | zscore |
| --- | --- | --- | --- | --- | --- | --- | --- | --- | --- | --- |
| BP | GO:0045766 | positive regulation of angiogenesis | 3/6 | 204/18670 | 2.5095E-05 | 0.00525622 | 0.00205745 | TERT/CCL11/IL1A | 3 | 1.73205081 |
| BP | GO:1904018 | positive regulation of vasculature development | 3/6 | 230/18670 | 3.5912E-05 | 0.00525622 | 0.00205745 | TERT/CCL11/IL1A | 3 | 1.73205081 |
| BP | GO:1901099 | negative regulation of signal transduction in absence of ligand | 2/6 | 36/18670 | 5.3962E-05 | 0.00525622 | 0.00205745 | TERT/IL1A | 2 | 1.41421356 |
| BP | GO:2001240 | negative regulation of extrinsic apoptotic signaling pathway in absence of ligand | 2/6 | 36/18670 | 5.3962E-05 | 0.00525622 | 0.00205745 | TERT/IL1A | 2 | 1.41421356 |
| BP | GO:0050768 | negative regulation of neurogenesis | 3/6 | 295/18670 | 7.5395E-05 | 0.00525622 | 0.00205745 | TERT/CCL11/SPP1 | 3 | 1.73205081 |
| BP | GO:0051961 | negative regulation of nervous system development | 3/6 | 315/18670 | 9.1628E-05 | 0.00525622 | 0.00205745 | TERT/CCL11/SPP1 | 3 | 1.73205081 |
| BP | GO:2001239 | regulation of extrinsic apoptotic signaling pathway in absence of ligand | 2/6 | 47/18670 | 9.2446E-05 | 0.00525622 | 0.00205745 | TERT/IL1A | 2 | 1.41421356 |
| BP | GO:0010721 | negative regulation of cell development | 3/6 | 344/18670 | 0.00011901 | 0.00592081 | 0.00231759 | TERT/CCL11/SPP1 | 3 | 1.73205081 |
| BP | GO:0045765 | regulation of angiogenesis | 3/6 | 383/18670 | 0.00016362 | 0.00722964 | 0.00282991 | TERT/CCL11/IL1A | 3 | 1.73205081 |
| BP | GO:0038034 | signal transduction in absence of ligand | 2/6 | 72/18670 | 0.00021781 | 0.00722964 | 0.00282991 | TERT/IL1A | 2 | 1.41421356 |
| BP | GO:0097192 | extrinsic apoptotic signaling pathway in absence of ligand | 2/6 | 72/18670 | 0.00021781 | 0.00722964 | 0.00282991 | TERT/IL1A | 2 | 1.41421356 |
| BP | GO:1901342 | regulation of vasculature development | 3/6 | 422/18670 | 0.00021798 | 0.00722964 | 0.00282991 | TERT/CCL11/IL1A | 3 | 1.73205081 |
| BP | GO:0014910 | regulation of smooth muscle cell migration | 2/6 | 84/18670 | 0.00029655 | 0.00907885 | 0.00355374 | TERT/PLAU | 2 | 1.41421356 |
| BP | GO:1903035 | negative regulation of response to wounding | 2/6 | 90/18670 | 0.0003404 | 0.00923367 | 0.00361434 | PLAU/SPP1 | 2 | 1.41421356 |
| BP | GO:0014909 | smooth muscle cell migration | 2/6 | 91/18670 | 0.000348 | 0.00923367 | 0.00361434 | TERT/PLAU | 2 | 1.41421356 |
| BP | GO:0014812 | muscle cell migration | 2/6 | 104/18670 | 0.00045432 | 0.01063641 | 0.00416342 | TERT/PLAU | 2 | 1.41421356 |
| BP | GO:2001237 | negative regulation of extrinsic apoptotic signaling pathway | 2/6 | 104/18670 | 0.00045432 | 0.01063641 | 0.00416342 | TERT/IL1A | 2 | 1.41421356 |
| BP | GO:2001236 | regulation of extrinsic apoptotic signaling pathway | 2/6 | 155/18670 | 0.00100501 | 0.02209912 | 0.00865028 | TERT/IL1A | 2 | 1.41421356 |
| BP | GO:0031214 | biomineral tissue development | 2/6 | 163/18670 | 0.00111051 | 0.02209912 | 0.00865028 | COL1A2/SPP1 | 2 | 1.41421356 |
| BP | GO:0045931 | positive regulation of mitotic cell cycle | 2/6 | 163/18670 | 0.00111051 | 0.02209912 | 0.00865028 | TERT/IL1A | 2 | 1.41421356 |
| BP | GO:0048771 | tissue remodeling | 2/6 | 179/18670 | 0.00133689 | 0.02313402 | 0.00905537 | SPP1/IL1A | 2 | 1.41421356 |
| BP | GO:0071347 | cellular response to interleukin-1 | 2/6 | 179/18670 | 0.00133689 | 0.02313402 | 0.00905537 | CCL11/IL1A | 2 | 1.41421356 |
| BP | GO:1903034 | regulation of response to wounding | 2/6 | 179/18670 | 0.00133689 | 0.02313402 | 0.00905537 | PLAU/SPP1 | 2 | 1.41421356 |
| BP | GO:0070555 | response to interleukin-1 | 2/6 | 207/18670 | 0.00178203 | 0.02955206 | 0.01156759 | CCL11/IL1A | 2 | 1.41421356 |
| BP | GO:0097191 | extrinsic apoptotic signaling pathway | 2/6 | 224/18670 | 0.00208244 | 0.03315237 | 0.01297686 | TERT/IL1A | 2 | 1.41421356 |
| BP | GO:2001234 | negative regulation of apoptotic signaling pathway | 2/6 | 230/18670 | 0.00219385 | 0.03358285 | 0.01314536 | TERT/IL1A | 2 | 1.41421356 |
| BP | GO:0060560 | developmental growth involved in morphogenesis | 2/6 | 235/18670 | 0.00228885 | 0.03373928 | 0.01320659 | CCL11/SPP1 | 2 | 1.41421356 |
| BP | GO:0090068 | positive regulation of cell cycle process | 2/6 | 298/18670 | 0.00365068 | 0.03773009 | 0.01476872 | TERT/IL1A | 2 | 1.41421356 |
| BP | GO:0007568 | aging | 2/6 | 321/18670 | 0.00422298 | 0.03773009 | 0.01476872 | TERT/CCL11 | 2 | 1.41421356 |
| BP | GO:0007596 | blood coagulation | 2/6 | 336/18670 | 0.00461754 | 0.03773009 | 0.01476872 | COL1A2/PLAU | 2 | 1.41421356 |
| BP | GO:0007599 | hemostasis | 2/6 | 341/18670 | 0.00475278 | 0.03773009 | 0.01476872 | COL1A2/PLAU | 2 | 1.41421356 |
| BP | GO:0050817 | coagulation | 2/6 | 342/18670 | 0.00478005 | 0.03773009 | 0.01476872 | COL1A2/PLAU | 2 | 1.41421356 |
| BP | GO:0001666 | response to hypoxia | 2/6 | 359/18670 | 0.00525493 | 0.03773009 | 0.01476872 | TERT/PLAU | 2 | 1.41421356 |
| BP | GO:0010038 | response to metal ion | 2/6 | 364/18670 | 0.00539864 | 0.03773009 | 0.01476872 | TERT/IL1A | 2 | 1.41421356 |
| BP | GO:0032102 | negative regulation of response to external stimulus | 2/6 | 365/18670 | 0.0054276 | 0.03773009 | 0.01476872 | PLAU/SPP1 | 2 | 1.41421356 |
| BP | GO:0030198 | extracellular matrix organization | 2/6 | 368/18670 | 0.00551493 | 0.03773009 | 0.01476872 | COL1A2/SPP1 | 2 | 1.41421356 |
| BP | GO:0032535 | regulation of cellular component size | 2/6 | 370/18670 | 0.00557352 | 0.03773009 | 0.01476872 | CCL11/SPP1 | 2 | 1.41421356 |
| BP | GO:0036293 | response to decreased oxygen levels | 2/6 | 370/18670 | 0.00557352 | 0.03773009 | 0.01476872 | TERT/PLAU | 2 | 1.41421356 |
| BP | GO:0045787 | positive regulation of cell cycle | 2/6 | 389/18670 | 0.0061446 | 0.03773009 | 0.01476872 | TERT/IL1A | 2 | 1.41421356 |
| BP | GO:0070482 | response to oxygen levels | 2/6 | 394/18670 | 0.00629924 | 0.03773009 | 0.01476872 | TERT/PLAU | 2 | 1.41421356 |
| BP | GO:0001503 | ossification | 2/6 | 398/18670 | 0.00642426 | 0.03773009 | 0.01476872 | COL1A2/SPP1 | 2 | 1.41421356 |
| BP | GO:1903532 | positive regulation of secretion by cell | 2/6 | 399/18670 | 0.00645569 | 0.03773009 | 0.01476872 | SPP1/IL1A | 2 | 1.41421356 |
| BP | GO:2001233 | regulation of apoptotic signaling pathway | 2/6 | 406/18670 | 0.00667774 | 0.03773009 | 0.01476872 | TERT/IL1A | 2 | 1.41421356 |
| BP | GO:0043062 | extracellular structure organization | 2/6 | 422/18670 | 0.00719849 | 0.03796208 | 0.01485953 | COL1A2/SPP1 | 2 | 1.41421356 |
| BP | GO:0051047 | positive regulation of secretion | 2/6 | 428/18670 | 0.00739848 | 0.03796208 | 0.01485953 | SPP1/IL1A | 2 | 1.41421356 |
| BP | GO:0060249 | anatomical structure homeostasis | 2/6 | 437/18670 | 0.00770327 | 0.03796208 | 0.01485953 | TERT/SPP1 | 2 | 1.41421356 |
| BP | GO:0022604 | regulation of cell morphogenesis | 2/6 | 484/18670 | 0.00938759 | 0.03860909 | 0.01511279 | CCL11/SPP1 | 2 | 1.41421356 |
| BP | GO:0048871 | multicellular organismal homeostasis | 2/6 | 485/18670 | 0.0094251 | 0.03860909 | 0.01511279 | SPP1/IL1A | 2 | 1.41421356 |
| BP | GO:0050900 | leukocyte migration | 2/6 | 499/18670 | 0.00995751 | 0.03923852 | 0.01535917 | COL1A2/CCL11 | 2 | 1.41421356 |
| CC | GO:0005788 | endoplasmic reticulum lumen | 2/6 | 309/19717 | 0.00352247 | 0.02369977 | 0.00831571 | COL1A2/SPP1 | 2 | 1.41421356 |
| MF | GO:0005125 | cytokine activity | 3/6 | 220/17697 | 3.6872E-05 | 0.00147488 | 0.00046575 | CCL11/SPP1/IL1A | 3 | 1.73205081 |
| MF | GO:0048018 | receptor ligand activity | 3/6 | 482/17697 | 0.0003777 | 0.00755403 | 0.00238548 | CCL11/SPP1/IL1A | 3 | 1.73205081 |
| MF | GO:0005126 | cytokine receptor binding | 2/6 | 286/17697 | 0.00374004 | 0.03249735 | 0.01026232 | CCL11/IL1A | 2 | 1.41421356 |
| KEGG | hsa05165 | Human papillomavirus infection | 3/6 | 331/8076 | 0.00124424 | 0.03306622 | 0.0255248 | TERT/COL1A2/SPP1 | 3 | 1.73205081 |
| KEGG | hsa04512 | ECM-receptor interaction | 2/6 | 88/8076 | 0.00171155 | 0.03306622 | 0.0255248 | COL1A2/SPP1 | 2 | 1.41421356 |
| KEGG | hsa04933 | AGE-RAGE signaling pathway in diabetic complications | 2/6 | 100/8076 | 0.00220441 | 0.03306622 | 0.0255248 | COL1A2/IL1A | 2 | 1.41421356 |
| KEGG | hsa04510 | Focal adhesion | 2/6 | 201/8076 | 0.00865534 | 0.08092916 | 0.06247163 | COL1A2/SPP1 | 2 | 1.41421356 |
| KEGG | hsa05205 | Proteoglycans in cancer | 2/6 | 205/8076 | 0.00899213 | 0.08092916 | 0.06247163 | COL1A2/PLAU | 2 | 1.41421356 |
