# Supplementary material for: Arecoline Is Associated With Inhibition of Cuproptosis and Proliferation of Cancer-Associated Fibroblasts in Oral Squamous Cell Carcinoma: A Potential Mechanism for Tumor Metastasis
Source: Front Oncol. 2022 Jul 7;12:925743. doi: 10.3389/fonc.2022.925743 (PMC9303015; doi:10.3389/fonc.2022.925743)
Supplement: Supplementary file 4 [file Table_3.docx]

| GO_ID | Simplified_ID |
| --- | --- |
| GOBP_FIBROBLAST_PROLIFERATION | Fibroproliferation 1 |
| GOBP_POSITIVE_REGULATION_OF_FIBROBLAST_PROLIFERATION | Fibroproliferation 2 |
| GOBP_NEGATIVE_REGULATION_OF_FIBROBLAST_PROLIFERATION | Fibroproliferation 3 |
| GOBP_RESPONSE_TO_FIBROBLAST_GROWTH_FACTOR | Fibroproliferation 4 |
| GOBP_FIBROBLAST_ACTIVATION | Fibroproliferation 5 |
| GOBP_FIBROBLAST_GROWTH_FACTOR_PRODUCTION | Fibroproliferation 6 |
| GOBP_NEGATIVE_REGULATION_OF_FIBROBLAST_GROWTH_FACTOR_PRODUCTION | Fibroproliferation 7 |
| GOBP_REGULATION_OF_CELL_CHEMOTAXIS_TO_FIBROBLAST_GROWTH_FACTOR | Fibroproliferation 8 |
| GOBP_NEGATIVE_REGULATION_OF_CELL_CHEMOTAXIS_TO_FIBROBLAST_GROWTH_FACTOR | Fibroproliferation 9 |
